# Conjugation of the 9-kDa Isoform of Granulysin with Liposomes Potentiates Its Cytotoxicity

**DOI:** 10.3390/ijms23158705

**Published:** 2022-08-05

**Authors:** Ruth Soler-Agesta, Patricia Guerrero-Ochoa, Joaquín Marco-Brualla, Raquel Ibáñez-Pérez, Isabel Marzo, Luis Martínez-Lostao, Alberto Anel

**Affiliations:** 1 Apoptosis, Immunity & Cancer Group, Department of Biochemistry and Molecular and Cell Biology, Faculty of Sciences, Aragón Health Research Institute (IIS Aragón), University of Zaragoza, 50009 Zaragoza, Spain; 2 Nanoscience Institute of Aragon (INA), 50018 Zaragoza, Spain; 3 Immunology Department, Clinical University Hospital “Lozano Blesa”, 50009 Zaragoza, Spain

**Keywords:** granulysin, lipid nanoparticles, acute lymphoid leukemia, apoptosis, immunotherapy

## Abstract

Nine kDa granulysin (GRNLY) is a human cytolytic protein secreted by cytotoxic T lymphocytes (CTL) and NK cells of the immune system whose demonstrated physiological function is the elimination of bacteria and parasites. In previous studies by our group, the anti-tumor capacity of recombinant granulysin was demonstrated, both in vitro and in vivo. In the present work, we developed lipid nanoparticles whose surfaces can bind recombinant granulysin through the formation of a complex of coordination between the histidine tail of the protein and Ni^2+^ provided by a chelating lipid in the liposome composition and termed them LUV-GRNLY, for granulysin-bound large unilamellar vesicles. The objective of this formulation is to increase the granulysin concentration at the site of contact with the target cell and to increase the cytotoxicity of the administered dose. The results obtained in this work indicate that recombinant granulysin binds to the surface of the liposome with high efficiency and that its cytotoxicity is significantly increased when it is in association with liposomes. In addition, it has been demonstrated that the main mechanism of death induced by both granulysin and LUV-GRNLY is apoptosis. Jurkat-shBak cells are resistant to GRNLY and also to LUV-GRNLY, showing that LUV-GRNLY uses the mitochondrial apoptotic pathway to induce cell death. On the other hand, we show that LUV-GRNLY induces the expression of the pro-apoptotic members of the Bcl-2 family Bim and especially PUMA, although it also induced the expression of anti-apoptotic Bcl-x_L_. In conclusion, we demonstrate that binding of GRNLY to the surfaces of liposomes clearly augments its cytotoxic potential, with cell death executed mainly by the mitochondrial apoptotic pathway.

## 1. Introduction

Granulysin (GRNLY) is a protein with two isoforms of 9 and 15 kDa molecular weight, respectively. The 9 kDa isoform is a cytolytic protein present in the granules of activated human cytotoxic T lymphocytes (CTL) and natural killer (NK) cells, while the 15 kDa isoform is rather an immune alarmin [1,2,3]. The antibacterial and antiparasitic functions of 9 kDa GRNLY have long been known, and they have been confirmed and expanded in recent studies [4,5,6]. We have shown previously that recombinant GRNLY is able to kill tumor cells in vitro using the mitochondrial apoptotic pathway [7,8,9]. On the other hand, there are abundant data that relate granulysin expression to an active antitumor immune response and to patient good prognosis [10,11,12,13]. Although mice do not express a granulysin homologue, transgenic mice that expressed human granulysin were generated, showing increased resistance to tumor development [14].

More recently, we demonstrated the efficacy of recombinant GRNLY in three xenotransplantation models of human tumors in athymic mice, the mammary adenocarcinoma MDA-MB-231, the multiple myeloma NCI-H929 and the melanoma UACC62 [15,16]. The intra-tumor administration of GRNLY in these models correlated with apoptosis induction in the tumor tissue and with prominent NK cell infiltration into the tumor mass, indicating that granulysin-induced tumor cell death in vivo could be immunogenic [15,16]. In an effort to target recombinant GRNLY specifically to tumors, we developed an immunotoxin by GRNLY gene fusion to the anti- carcinoembryonic antigen (CEA/CEACAM5) single chain Fv antibody fragment MFE23. Systemic administration of the immunotoxin demonstrated a decrease in tumor growth in a CEA+ tumor-bearing mouse model, whereas GRNLY did not exhibit a therapeutic effect [17]. Other GRNLY-based immunotoxins directed against the MUC1-Tn tumor antigen have been applied, demonstrating in vitro and in vivo efficacy in pre-clinical models [18,19]. These immunotoxins could have a broader application to different solid tumors and leukemia than the anti-CEA immunotoxin [20]. 

Considering the perspective of the therapeutic use of GRNLY in a broader spectrum of cancers, it is important to develop strategies that allow its systemic administration. This will require vehicles that increase GRNLY ability to access the tumor while preserving or increasing its bioactivity. In previous experiences using large unilamellar vesicles (LUVs), a drug delivery vehicle that consists of a phospholipid bilayer containing an inner, aqueous pocket [21], our group demonstrated greater cytotoxicity against tumors using LUV conjugated with TNF-related apoptosis-inducing ligand (TRAIL) compared with TRAIL in soluble phase [22,23,24]. We also demonstrated both in vitro and in vivo that this anti-tumor effect was increased if doxorubicin was encapsulated in the TRAIL-decorated LUVs [25].

In the present study, we generated LUV conjugated with GRNLY (LUV-GRNLY) and tested its antitumor efficacy and mechanism of tumor cell death induction. Results demonstrate that our hypothesis of a possible increase in GRNLY cytotoxicity by binding to liposomes was correct, and offer new perspectives for this novel cytotoxic formulation based on a human protein.

## 2. Results

### 2.1. Production and Purification of the Recombinant 9 kDa GRNLY in Pichia Pastoris

**Figure 1:**
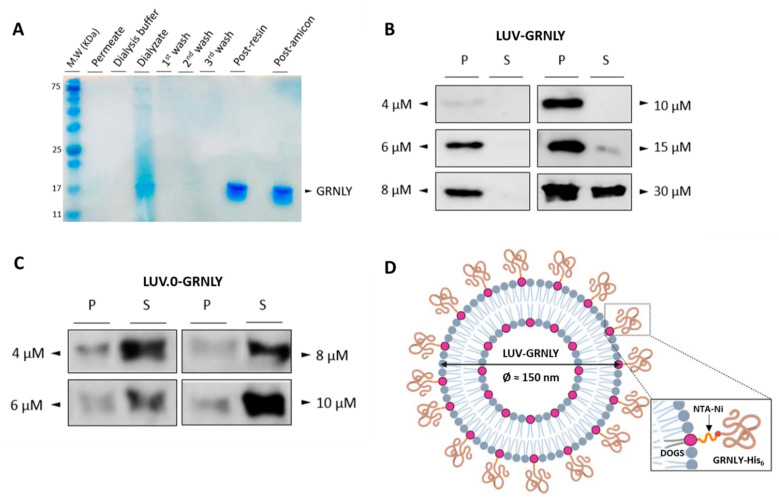
(**A**) Analysis of the purification process using SDS-PAGE polyacrylamide 15% gel stained with *Coomassie* blue. (**B**) Western-blot analysis of ultracentrifugated pellets (P) and supernatant (S) fractions from LUV-GRNLY using an anti-His antibody. (**C**) Western blot of ultracentrifugated pellets and supernatant fractions from LUV.0-GRNLY using an anti-His antibody. (**D**) Schematic representation of the interaction between GRNLY-His_6_ and liposome surface through DOGS-NTA-Ni.

As described in Materials and Methods, granulysin was subcloned into the pPICZαA vector for its production in Pichia pastoris. The recombinant protein was secreted in yeast culture supernatant and purified using a gravity-flow column containing an Ni+2-NTA resin. During the purification process, the presence of granulysin was controlled by recollecting samples in each step (concentrated, dialysis, resin washes, post-resin and post-amicon fractions) and resolved in an SDS-PAGE 15% polyacrylamide gel stained with Coomassie blue. The migration pattern of the dialysate fraction, as shown in Figure 1A, revealed several bands that correspond to a mixture of proteins produced by Pichia pastoris. Discarded fractions such as the permeate, the dialysis buffer and the resin washes showed that there was no loss of protein at the granulysin molecular weight. Finally, the migration pattern of post-resin and post-amicon fractions showed a single concentrated band that was consistent with the molecular weight of glycosylated recombinant granulysin produced in Pichia pastoris [17,26].

### 2.2. GRNLY Binds Efficiently to LUV Surface through the DOGS-NTA-Ni Lipid

In order to optimize the binding of GRNLY-His6 to DOGS-NTA-Ni in the liposome, several concentrations of granulysin, between 4 and 30 μM, were incubated with liposomes, ultracentrifugated, and pellet and supernatant collected separately. The presence of the recombinant protein in each fraction was assessed by Western blot using an antibody against the histidine-tag motif. As shown in Figure 1B, from 4 to 10 µM, all of the recombinant protein was found only in the pellet fraction, indicating that all of the protein was bound to the liposome surfaces. Nevertheless, at 15 µM, a small portion of the protein remained free in the supernatant, indicating that the surfaces of the liposomes began to be saturated. Finally, at 30 µM, a significant protein band appeared in the supernatant fraction, showing the full saturation of the liposome surface. In the following experiments, the stock of LUV-GRNLY was prepared with a 15 µM concentration of recombinant GRNLY.

To confirm that the recombinant protein binds to the liposome surface through the lipid DOGS-NTA-Ni, increasing granulysin concentrations were incubated with LUV.0, a variant liposome formulation without DOGS-NTA-Ni. As shown in Figure 1C, recombinant granulysin was found mainly in the supernatant fractions for all tested concentrations. A small portion of the protein was also found in the pellet, showing that granulysin binds partially to the lipid membrane. A schematic representation of LUV-GRNLY once synthesized is shown in Figure 1D.

### 2.3. In Vitro Cytotoxic Effect of LUV-GRNLY on the Jurkat Cell Line

**Figure 2:**
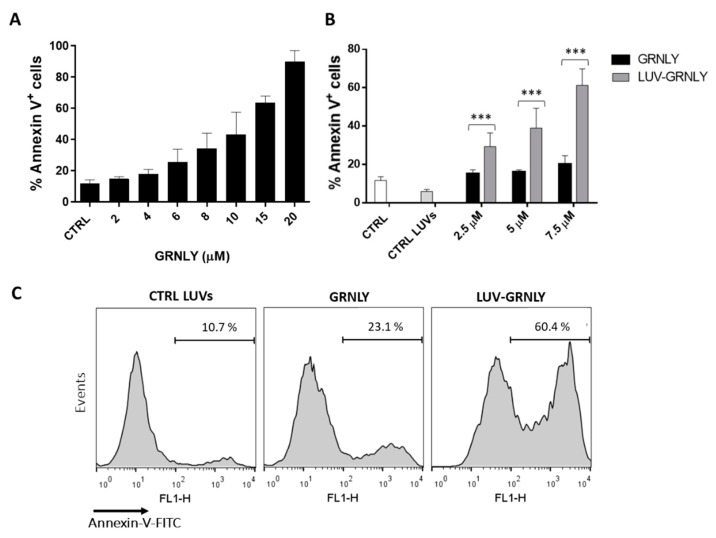
In vitro cytotoxicity of GRNLY and LUV-GRNLY against Jurkat cell line. (**A**) Jurkat cells were incubated with increasing concentrations of GRNLY for 24 h. Cell death was determined by detection on phosphatidylserine exposure (PS) by staining with annexin-V-FITC using flow cytometry. (**B**) Jurkat cells were incubated with increasing concentrations of GRNLY and LUV-GRNLY (2.5, 5 and 7.5 µM) for 24 h, and cell death was determined by flow cytometry as previously described. The results are expressed as the mean ± SD of 3 independent experiments. *** *p* < 0.001. (**C**) Representative flow cytometry histograms corresponding to one of the experiments performed, showing the Annexin-V-FITC staining data (FL1-H channel) on Jurkat cells treated with control LUVs, with 7.5 µM GRNLY, or with 7.5 µM LUV-GRNLY, as indicated.

We then determined the in vitro cytotoxicity of LUV-GRNLY against the Jurkat acute lymphocytic leukemia cell line and compared it with soluble GRNLY using flow cytometry. It has been described previously that soluble GRNLY is cytotoxic against Jurkat cells and that it is cytotoxic against a large panel of cancer cells [7,8]. We confirmed this observation in the dose–response assays shown in Figure 2A, showing an LC50 of around 10 µM, compatible with previous data using GRNLY produced in Pichia pastoris [17]. Besides that, as shown in Figure 2B, we demonstrated that LUV-GRNLY exhibited a higher cytotoxicity than GRNLY in soluble phase, reducing the LC_50_ by at least two-fold. In addition, the mortality rate of cells that were treated with liposomes alone was not significant compared to the control. Therefore, we concluded that the cytotoxicity of LUV-GRNLY is attributed to the formula by itself, discarding the possibility that liposomes could be toxic at this dose.

### 2.4. Analysis of the Mechanism of Cell Death Induced by GRNLY and LUV-GRNLY in Jurkat Cells

**Figure 3:**
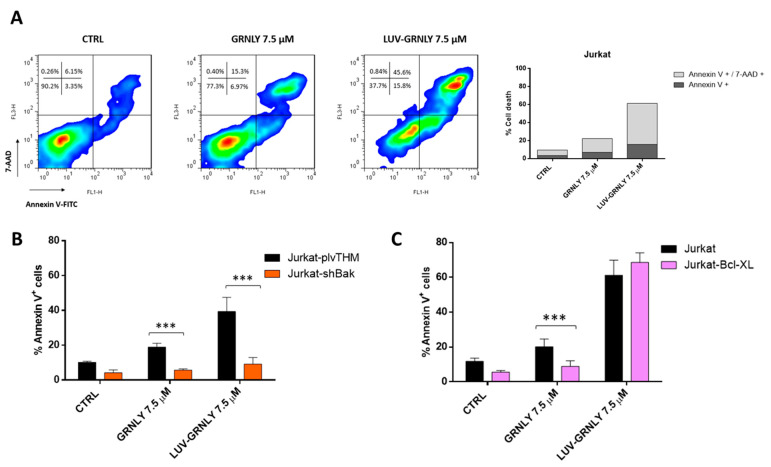
Analysis of the mechanism of cell death induced by GRNLY and LUV-GRNLY. (**A**) Jurkat cells were treated with 7.5 µM of GRNLY or LUV-GRNLY for 24 h, respectively. Then, cells were simultaneously stained with annexin-V-FITC and 7-AAD and were analyzed using flow cytometry. The dot-plots (**upper**, **left panel**) represent the evolution of treated cell population compared to the control. The values shown correspond to a percentage of cells in each quadrant. The upper right figure corresponds to a graphical representation of obtained data. Data shown are representative of four different experiments. (**B**,**C**) Jurkat and mutant cells (Jurkat-Bcl-x_L_, Jurkat-plvTHM and Jurkat-shBak) were treated with 7.5 µM of GRNLY or LUV-GRNLY, respectively, for 24 h. Cell death was determined by exposure of PS by staining with annexin-V-APC and analyzing by flow cytometry. The results are expressed as the mean ± SD of 3 independent experiments. *** *p* < 0.001.

To further investigate the mechanism of cell death induced by GRNLY and LUV-GRNLY, we carried out flow cytometry experiments in which cells were stained at the same time with annexin-V and 7-AAD. The presence of a population of cells that were positive for annexin-V staining and still negative for 7-AAD staining was indicative of an apoptotic type of cell death, although at long times of in vitro exposure to toxic agents, cells were found to be positive for both markers in a secondary necrosis process. The single positive population could be detected after treatment with 7.5 µM GRNLY for 24 h, constituting 7% of the total population; at this time, the double positive population represented 15% (Figure 3A). This observation indicated the presence of an apoptotic type of cell death, in agreement with previous observations made with this cell line [7]. Cells treated with 7.5 µM LUV-GRNLY for 24 h showed the same staining characteristics: we observed again the single positive population (16% of the total population) and the double positive population (45%). Confirming data shown in Figure 2, the total percentage of cell death induced by LUV-GRNLY was higher (61.4% vs. 22.3%). Thus, we showed that both GRNLY and LUV-GRNLY induce a rather apoptotic type of cell death, although LUV-GRNLY was more cytotoxic than GRNLY alone. 

Previous work has shown that recombinant 9 kDa GRNLY was able to induce apoptosis mainly through the mitochondrial apoptotic pathway [7]. In order to assess if LUV-GRNLY followed the same type of mechanism, we carried-out cytotoxicity experiments with modified Jurkat cell lines: Jurkat-Bcl-x_L_, which over-expressed the anti-apoptotic protein Bcl-x_L,_ and Jurkat-shBak, which did not express the pro-apoptotic protein Bak. Jurkat cells are naturally deficient in the expression of the pro-apoptotic protein Bax and thus, Jurkat-shBak is a double Bax and Bak mutant and, in consequence, is resistant to the intrinsic apoptosis pathway [22].

Indeed, the silencing of the pro-apoptotic protein Bak in the mutant Jurkat-shBak cell line was able to fully inhibit cell death induced by both treatments compared with its positive control (Jurkat plvTHM), indicating that GRNLY and LUV-GRNLY-induced cell death depends on the presence of the pro-apoptotic proteins Bak and Bax and proceeds through the mitochondrial apoptosis pathway (Figure 3B). However, over-expression of the anti-apoptotic protein Bcl-xL in mutant cells provides protection against granulysin, but not against LUV-GRNLY (Figure 3C), suggesting that the LUV-GRNLY-induced cell death signal is more powerful than that of GRNLY alone.

### 2.5. GRNLY and LUV-GRNLY Treatments Increase PUMA, Bim and Bcl-x_L_ Expression

The mitochondrial apoptotic pathway or intrinsic cell death pathway is initiated by the induction of expression of BH3-only pro-apoptotic proteins of the Bcl-2 superfamily, such as Bim and PUMA [27]. Since the mitochondrial apoptotic pathway is implicated in GRNLY- or LUV-GRNLY-induced cell death, we next studied if GRNLY or LUV-GRNLY was able to induce Bim or PUMA expression in Jurkat cells.

**Figure 4:**
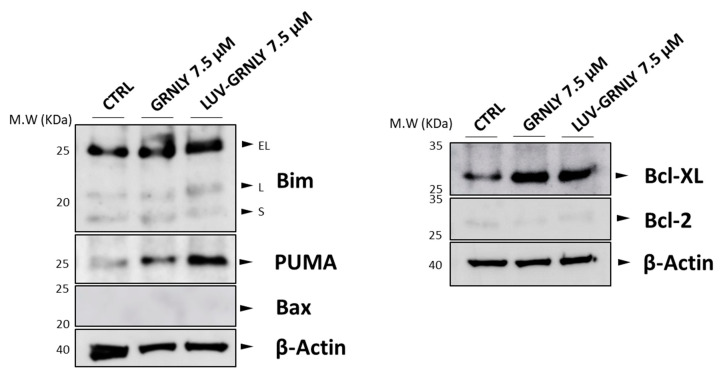
Expression levels of proteins from the Bcl-2 family upon treatment with GRNLY or LUV-GRNLY. The expression of Bim, PUMA, Bax, Bcl-x_L_ or Bcl-2 in Jurkat cells treated with 7.5 µM of GRNLY or LUV-GRNLY for 24 h was analyzed by Western blot using specific antibodies. Cell lysates (5 × 10^6^ cells) were separated by SDS-PAGE 12% polyacrylamide gel, transferred to PVDF membranes and analyzed by Western blot using specific antibodies. Levels of β-actin were used as a control of protein loading.

As shown in Figure 4, we observed that GRNLY clearly induced PUMA expression, while the induction of Bim was rather limited. However, LUV-GRNLY clearly induced the expression of both Bim and PUMA, further demonstrating that it is a more potent cellular stressor and cell death inductor than GRNLY alone. We also confirmed the absence of expression of Bax in these cells, which is not induced by either GRNLY or LUV-GRNLY. Remarkably, both GRNLY and LUV-GRNLY also induced the expression of the anti-apoptotic member of the Bcl-2 family Bcl-x_L_, probably as a mechanism of cell defense. Finally, the low levels of Bcl-2 were not affected by the treatments.

### 2.6. Toxicity against PBMC and T Cell Blasts from Healthy Donors

**Figure 5:**
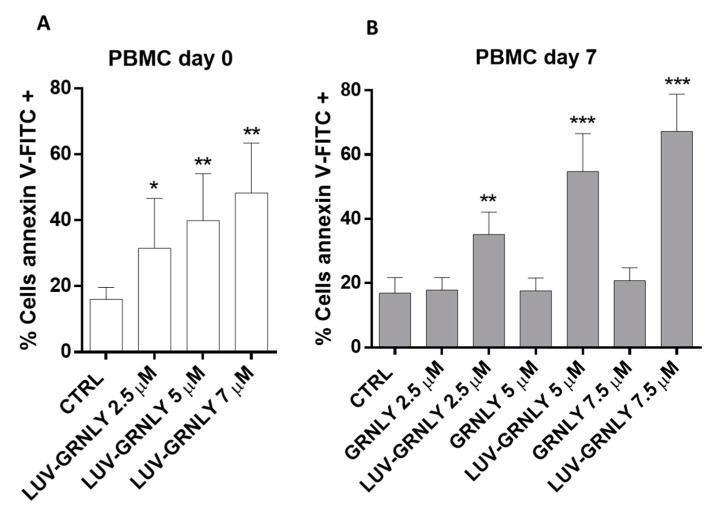
Toxicity of GRNLY or LUV-GRNLY towards fresh PBMC or 7-day T cell blasts obtained from healthy donors. (**A**) Freshly isolated PBMCs from healthy donors were treated for 24 h with increasing concentrations of LUV-GRNLY, as indicated. (**B**) Seven-day T cell blasts from the same healthy donors were treated for 24 h with increasing concentrations of GRNLY or LUV-GRNLY, as indicated. Then, cells were stained with annexin-V-FITC and analyzed using flow cytometry. * *p* < 0.5; ** *p* < 0.01; *** *p* < 0.001.

We have previously shown that recombinant GRNLY, while being toxic against tumor cells, was not active against fresh PBMC obtained from healthy donors [7]. We performed a similar experiment using LUV-GRNLY and found that it was toxic against freshly isolated PBMC (Figure 5A). In addition, we also demonstrated that, while GRNLY was not toxic against day-7 T cell blasts, at least until a concentration of 7.5 µM, LUV-GRNLY proved to be highly toxic (Figure 5B).

## 3. Discussion

Our previous studies using recombinant TRAIL anchored to the surface of liposomes (LUV-TRAIL) demonstrated that this new formulation was much more potent than soluble TRAIL both in different tumor types both in vitro and in vivo [22,23,24]. Although the liposomes for disease treatment have been extensively used in different diseases, liposomes have also been used as a vehicle to encapsulate drugs [28,29,30], or more recently, mRNA in vaccine development [31]. However, attachment of bioactive compounds on the liposome surface has been a less explored therapeutic strategy. In this line, the attachment of TRAIL to the surface of liposomes, an improvement proposed by our group, resulted in increased anti-tumor bioactivity, and in fact tried to mimic the natural way of secretion of TRAIL and FasL bound to exosomes, described initially in human T cell blasts [32,33,34]. Liposome-based treatments show several advantages: biocompatibility, increased efficacy and stability of encapsulated agents as well as reduction of their toxicity, and provide selective passive targeting to tumor tissues. In fact, liposome-based formulations would take advantage of the well-known enhancing permeability and retention (EPR) effect. According to the EPR effect, nanoparticles in the 100 nm diameter range spontaneously localize in tumor sites by extravasation in the leaky blood capillary system irrigating the tumor [35,36].

Based on our previous work, our working hypothesis was that the association of GRNLY with liposomes could increase protein concentration at the site of contact with the target cell, and, therefore, it could potentiate the cytotoxicity of the administered dose. This approach could lead to a reduction in the dose needed to attain anti-tumor effects. 

Analysis of interaction of recombinant GRNLY containing a histidine tag with LUV demonstrated that the attachment of GRNLY using a novel Ni^2+^-NTA–containing lipid in the liposome composition was extremely efficient, with recoveries close to 100% at 10–15 µM GRNLY concentrations. The chemical basis for conjugation of GRNLY to liposome surface is the formation of a stable coordination complex between the histidine tag of the recombinant GRNLY and the Ni^2+^ cation of DOG-NTA-Ni. When higher concentrations of recombinant GRNLY (more than 15 µM) were used, the liposome surface was saturated, not admitting higher loading of more GRNLY molecules. In any case, assuming that the binding efficiency of GRNLY to liposomes surface is 100%, we estimated that each liposome should harbor around 1438 GRNLY molecules on its surface when using 15 µM GRNLY.

We have shown that our hypothesis was correct, since the cytotoxicity of LUV-GRNLY is higher than that of the same dose of GRNLY in the soluble phase, reducing the LC50 against Jurkat cells between 2- and 3-fold. A possible explanation for the increased cytotoxicity of LUV-GRNLY, as mentioned above, is that association of GRNLY to liposome surface increases the local concentration of recombinant protein that can interact with cell membrane surface, in turn increasing its bioactivity. 

We have also investigated the cell death mechanisms induced by LUV-GRNLY, finding that it was similar to that previously described for recombinant GRNLY, mainly depending on the mitochondrial apoptotic pathway. Remarkably, we found that although LUV-GRNLY-induced cell death was abrogated in the absence of Bax and Bak expression in Jurkat-shBak cells, LUV-GRNLY was still effective against Jurkat cells over-expressing Bcl-x_L_, while GRNLY was not. This is an interesting observation regarding the possible clinical application of our formulation, since in many instances, tumor cells resistant to conventional chemotherapy over-express anti-apoptotic members of the Bcl-2 family [37]. This, in fact, has led to the recent introduction, into the antitumor arsenal, of BH3 mimetics that counteract these types of resistance, such as venetoclax, which has been approved for the treatment of a variety of hematological malignancies [38,39]. 

In a previous study, we showed that although recombinant GRNLY was toxic against hematological tumor cells, it was not active against fresh PBMCs obtained from healthy donors [7]. This indicated that GRNLY had some tumor selectivity, probably dependent on the different membrane lipid compositions of normal and tumor cells [20,40], something that has been confirmed by its absence of in vivo toxicity [15]. However, LUV-GRNLY proved to be toxic against freshly isolated PBMCs and also against T cell blasts, indicating that increases in its bioactivity could also lead to undesired secondary effects. Although one of the advantages of liposomal formulations is the reduction in toxic effects of the bioactive molecules, mainly for encapsulated drugs, a crucial step in the generation of functionalized liposomes is the final concentration of the bioactive compounds either encapsulated or attached to their surface, to avoid their adverse effects. One possibility that could explain the toxicity of LUV-GRNLY against healthy cells would be that the liposome surface is saturated with GRNLY molecules. In this line, previous studies by our group have demonstrated that reducing the concentration of bioactive compounds in liposomal formulations reduced their toxic adverse effects while retaining their cytotoxic effects against tumor cells [25]. Therefore, further studies generating LUV-GRNLY with lower amounts of GRNLY on their surface could show that this non-saturated LUV-GRNLY is able to retain its anti-tumoral effects while reducing its toxic effects against healthy cells. 

## 4. Materials and Methods

### 4.1. Cell Culture

The acute T cell leukemia Jurkat cell line was purchased from ATCC. Cells over-expressing the anti-apoptotic protein Bcl-xL (Jurkat-Bcl-x_L_) and cells lacking the expression of the pro-apoptotic protein Bak (Jurkat-shBak) were generated in our laboratory using a lentiviral system, as previously described [22]. These cell lines are completely resistant to doxorubicin, a drug that kills cells through the mitochondrial apoptotic pathway [22]. Cell lines were routinely cultured in RPMI 1640 medium with GlutaMAX (Life Technologies, Paisley, UK) supplemented with 10% fetal calf serum (FCS), penicillin (1000 U/mL) and streptomycin (10 mg/mL) (PanBiotech, Aidenbach, Germany) at 37 °C and 5% CO_2_ using standard procedures. Human PBMCs from healthy donors were obtained by Ficoll density centrifugation from Leukopacks provided by the “Banco de Sangre y Tejidos de Aragón”. T-cell blasts were generated from PBMCs from the same donors by stimulation with PHA at 10 µg/mL overnight, with posterior washing and culture for at least 7 days in the presence of 30 IU/mL of IL-2, with medium changes each 48 h, as described in [41]. The use of human samples was approved by the “Comité Etico de la Investigación de Aragón” (CEICA) and written informed consent was obtained from each donor.

### 4.2. Expression and Purification of 9 kDa Granulysin

Synthetic genes encoding 6× His-tagged 9 kDa granulysin, kindly provided by Dr. Alan M. Krensky, were synthesized by Geneart GmbH (Thermo Fisher Scientific Regensburg, Regensburg, Germany) and subcloned between Cla I and XbaI sites into pCR3.1, resulting in pCR3.1-GRNLY, as indicated in [17]. Then, 293 T cells were transiently transfected with the vector using calcium phosphate, and supernatants were analyzed for protein expression. The Cla I/XbaI -digested fragments of pCR3.1-GRNLY were then ligated into the Cla I/XbaI digested backbone of plasmid pPICZα A to obtain pPICZα A-GRNLY. The plasmid was then amplified in *E.coli* and isolated by NucleoSpin^®^ Plasmid EasyPure (Macherey-Nagel, Düren, Germany). Plasmids were linearized with SacI (Takara) and purified by Ilustra™ GFX™ PCR DNA and Gel Band Purification kit (GE Healthcare). 

Competent cultures of Pichia pastoris were then transfected with the pPICZαA vector containing recombinant 9 kDa granulysin and expanded in aseptic YPD agar (Formedium, Hunstanton, UK) plate supplemented with zeocin (100 μg/mL) (Invitrogen, Carlsbad, CA, USA) for 3 days, as indicated in [17]. One of the colonies was transferred in a BMGY medium (10 g/L yeast extract; 20 g/L de peptone; 100 mM potassium phosphate pH = 6.0; 13.4 g/L yeast base; 1 ml/L glycerol and 0.4 mg/L biotin, all purchased from Formedium, Hunstanton, UK) and was incubated at 30 °C and 250 rpm for 24 h. Next, culture medium was exchanged with BMMY medium (10 g/L yeast extract; 20 g/L peptone; 100 mM phosphate buffer pH = 6.0; 13.4 g/L yeast base; 0.5 mL/L methanol y 0.4 mg/L de biotin, all purchased from Formedium, Hunstanton, UK) by centrifugation at 4400 rpm for 25 min. Then, yeast culture was incubated at 18 °C with agitation for 48 h. Every 24 h, methanol was added in a weight ratio of 1:100 in order to induce granulysin expression. For protein purification, the supernatant was filtered through 0.45 and 0.22 μm filters (Merck Millipore, Tullagreen, Ireland) and concentrated with a Pellicon XL Ultracel 50 cm^2^ cassette (Merck Millipore, Jaffrey, NH, USA) connected to a vacuum pump. The concentrated supernatant was dialyzed overnight in a dialysis buffer (300 mM NaCl; 50 mM Tris-HCl y 20 mM imidazole, pH = 7.8) at 4 °C. Next, the dialyzed fraction was incubated with Ni-NTA resin (Qiagen, Redwood City, CA, USA) at 4 °C for 2 h, gently shaking. The resin was washed 3 times and then transferred in a gravity-flow column in which the protein was eluted with an elution buffer composed of 400 mM imidazole, 300 mM NaCl and 50 mM de Tris- HCl, pH 7.4. The imidazole was exchanged with KHE buffer using Amicon^®^ filters (Merck Millipore, Tullagreen, Ireland). Protein concentration was measured using a BCA assay (Thermo Fisher, Rockford, IL, USA), and its purity was checked by SDS-PAGE in 15% polyacrylamide gel, Coomassie blue staining and immunoblot incubated with specific anti-His antibody (Genescript, Piscataway, NJ, USA). Finally, the protein was sterilized through a 0.22 μm filter and was stored at 4 °C. The stability of the protein was maintained at 4 °C for at least 1 month.

### 4.3. Large Unilamellar Vesicle (LUV) Synthesis and Preparation of Lipid Nanoparticles Coated with 9 kDa Recombinant Granulysin (LUV-GRNLY)

The generation of large unilamellar vesicles (LUVs) was achieved by preparing a mixture of phosphatidylcholine, sphingomyelin, cholesterol, 1,2-dioleoyl-sn-glycero-3[N-(5-amino-1-carboxypentyl)-iminodiacetic acid]succinyl (DOGS-NTA-Ni) and polyethylene glycol (PEG) in a weight ratio of 55:30:10:5:5, previously dissolved in chloroform/methanol (2:1), as originally described in [42]. All lipids were purchased from Avanti Polar Lipids, Alabama, AL, USA. The chloroform and methanol were removed under nitrogen gas for 10 min and then under vacuum conditions at 45 °C for 6 h. After that, lipids were dissolved in a KHE buffer, and the aliquots were frozen overnight (−20 °C). The next day, aliquots were thawed and extruded at least 10 times through a polycarbonate filter with 200 nm pore diameter (Whatman, Maidstone, UK) using an extruder (Northern Lipids, Burnaby, BC, Canada), to generate classical large unilamellar vesicles (LUVs), which mimic the composition of natural exosomes. Finally, LUVs were incubated with 9 KDa granulysin in KHE buffer for 30 min at 37 °C with gentle shaking (800 rpm). During this step, the polyhistidine tail of granulysin established a complex of coordination with Ni2+ provided by a chelating lipid (DOGS-NTA-Ni) in the liposome composition that remained attached permanently. LUV-GRNLY could be stored at 4 °C for 1 month without losing its bioactivity.

### 4.4. Optimization Procedure for LUV-GRNLY Synthesis; Binding and Affinity Assays

In order to examine the affinity of the histidine-tagged granulysin to DOGS-NTA-Ni and the protein load that each liposome can harbor on its surface, different concentrations of granulysin (4, 6, 8, 10, 15 y 30 μM) were incubated with liposomes in KHE buffer for 30 min at 37 °C and 800 rpm. Every mixture was ultracentrifugated for 5 h at 14,000 rpm in an L8-60M Ultracentrifuge (Beckman and Coulter). The supernatant was separated from the pellet and dissolved in the same volume of KHE buffer. Finally, the presence of protein in both fractions was analyzed by Western-blot technique using an anti-His antibody (GenScript, Piscataway, NJ, USA) and Coomassie blue staining in 15% SDS-PAGE gel electrophoresis. Equally, in order to demonstrate that granulysin binds to liposome through DOGS-NTA-NI, liposomes without DOGS-NTA-Ni (LUV.0) were synthetized and incubated with increased concentrations of granulysin. The protein union to the surface was assessed by reproducing the same procedure.

### 4.5. Estimation of the Number of GRNLY Molecules per Liposome 

Assuming that 100% of the generated liposomes are unilamellar and that their diameters are approximately 150 nm, the lipid number that composes one liposome can be calculated with the following equation:

$$N{}^{\circ}\;lipo\;TOT=\frac{\left[4\pi {\left(\frac{d}{2}\right)}^{2}+4\pi \;{\left[\frac{d}{2}-h\right]}^{2}\right]}{a}$$

where:

*d* is the diameter of the liposome (150 nm)

*h* is the thickness of the liposome (5 nm)

*a* is the area of the hydrophilic heads of phospholipids (0.55 nm^2^)

From the equation, we calculated that one liposome is composed of approximately 240,000 lipid molecules, of which 5% correspond to DOGS-NTA-Ni. Therefore, the number of DOGS-NTA-Ni molecules that would be theoretically available for binding to the protein was 12,000.

For a final concentration of lipids/mL of 2.5 mM, we calculated that the total number of liposomes/mL would be 6.26 × 10^12^. Finally, if we prepared an aliquot of 15 µM of LUV-GRNLY, the number of GRNLY molecules in 1 mL would be 9 × 10^15^. Assuming that the bonding efficiency of GRNLY to liposome surfaces is 100%, we estimated that each liposome should harbor around 1438 GRNLY molecules on its surface.

### 4.6. In Vitro Cytotoxicity Assays—Apoptosis Quantification by Flow Cytometry

Cytotoxicity assays were carried-out as follows: 50 µL aliquots of 3 × 10^4^ cells were seeded per well in 96-well plates and 50 µL aliquots of increasing concentrations of GRNLY or LUV-GRNLY were added and incubated for 24 h at 37 °C. For controls wells the same volume of KHE buffer was added. Cell death was analyzed using a FACScalibur flow cytometer (BD, Biosciences, Franklin Lakes, NJ, USA) after incubation with annexin-V-FITC or APC (BD Biosciences) in annexin binding buffer (140 mM NaCl, 2.5 mM CaCl_2_, 10 mM HEPES/NaOH, pH 7.4) for 20 min. The 7-AAD staining (BD Biosciences) was also performed in some experiments. The data obtained was analyzed using FlowJo 0.7 (Tree star Inc., San Francisco, CA, USA).

### 4.7. Western-Blot Analysis

A total of 5 × 10^6^ cells were lysed with 100 µL of a lysis buffer 1× (1% Triton-X-100; 150 mM NaCl; 50 mM Tris/HCl pH 7.6; 10% *v/v* glycerol; 1mM EDTA; 1mM sodium orthovanadate; 10 mM sodium pyrophosphate; 10 μg/mL leupeptin; 10 mM sodium fluoride; 1 mM methyl phenyl sulfide, Sigma, St. Louis, MO, USA) for 30 min in ice. The mixture was centrifuged at 12,000 rpm for 20 min at 4 °C. The protein concentration in supernatant was analyzed using a BCA assay (Thermo Fisher, Rockford, IL, USA) and was mixed with lysis buffer 3× (SDS; 150 mM Tris/HCl; 0.3 mM sodium molybdate; 30% *v/v* glycerol; 30 mM sodium pyrophosphate; 30 mM sodium fluoride; 0.006% *w/v* bromophenol blue; 30% *v/v* 2-mercaptoethanol, all purchased from Sigma, St. Louis, MO, USA). Protein separation was performed using SDS-PAGE 12% polyacrylamide gel, and then proteins were transferred to nitrocellulose membranes using a semi dry electro transfer (Biorad, Alcobendas, Spain). Membranes were blocked with TBS-T buffer (Tris/HCl 10 mM, pH 8; NaCl 0.12 M; Tween-20 0.1%, thimerosal 0.1 g/L, Sigma, St. Louis, MO, USA) containing 5% skimmed milk. Protein detection was performed by the Western-blot technique, using specific antibodies against PUMA (Novus International, St. Louis, MO, USA), Bax, Bim, Bcl-x_L_ (Cell Signaling, Danvers, MA, USA), Bcl-2 (Santa Cruz, Dallas, USA) and β-actin (Cell Signaling, Danvers, MA, USA) that were incubated overnight at 4 °C with agitation. Anti-rabbit and anti-mouse secondary antibodies labeled with peroxidase (Sigma, St. Louis, MO, USA) were incubated for 1 h at room temperature, gently shaking. Proteins were revealed with the reagent Pierce ELC Western Blotting Substrate (Thermo Scientific, Rockford, IL, USA) using Amersham Imager 680 (GE Healthcare Life Sciences).

### 4.8. Statistical Analysis

Computer-based statistical analysis was performed using the GraphPad Prism program (GraphPad Software Inc., San Diego, CA, USA). For quantitative variables, results are shown as median ± standard deviation (SD). Statistical significance was evaluated using Student t test, and differences were considered significant when *p* < 0.05.

## Data Availability

The data presented in this study are available on request from the corresponding author.

## References

[1] Clayberger C., Finn M.W., Wang T., Saini R., Wilson C., Barr V.A., Sabatino M., Castiello L., Stroncek D., Krensky A.M. (2012). 15 kDa Granulysin Causes Differentiation of Monocytes to Dendritic Cells but Lacks Cytotoxic Activity. J. Immunol..

[2] Clayberger C., Krensky A.M. (2003). Granulysin. Curr. Opin. Immunol..

[3] Peña S.V., Hanson D.A., Carr B.A., Goralski T.J., Krensky A.M. (1997). Processing, subcellular localization, and function of 519 (granulysin), a human late T cell activation molecule with homology to small, lytic, granule proteins. J. Immunol..

[4] Crespo Â., Mulik S., Dotiwala F., Ansara J., Sen Santara S., Ingersoll K., Ovies C., Junqueira C., Tilburgs T., Strominger J. (2020). Decidual NK Cells Transfer Granulysin to Selectively Kill Bacteria in Trophoblasts. Cell.

[5] Dotiwala F., Mulik S., Polidoro R., Ansara J., Burleigh B., Walch M., Gazzinelli R., Lieberman J. (2016). Killer lymphocytes use granulysin, perforin and granzymes to kill intracellular parasites. Nat. Med..

[6] Stenger S., Hanson D.A., Teitelbaum R., Dewan P., Niazi K.R., Froelich C.J., Ganz T., Thoma-Uszynski S., Melián A., Bogdan C. (1998). An antimicrobial activity of cytolytic T cells mediated by granulysin. Science.

[7] Aporta A., Catalán E., Galán-Malo P., Ramírez-Labrada A., Pérez M., Azaceta G., Palomera L., Naval J., Marzo I., Pardo J. (2014). Granulysin induces apoptotic cell death and cleavage of the autophagy regulator Atg5 in human hematological tumors. Biochem. Pharmacol..

[8] Gamen S., Hanson D.A., Kaspar A., Naval J., Krensky A.M., Anel A. (1998). Granulysin-induced apoptosis. I. Involvement of at least two distinct pathways. J. Immunol..

[9] Martinez-Lostao L., de Miguel D., Al-Wasaby S., Gallego-Lleyda A., Anel A. (2015). Death ligands and granulysin: Mechanisms of tumor cell death induction and therapeutic opportunities. Immunotherapy.

[10] Kishi A., Takamori Y., Ogawa K., Takano S., Tomita S., Tanigawa M., Niman M., Kishida T., Fujita S. (2002). Differential expression of granulysin and perforin by NK cells in cancer patients and correlation of impaired granulysin expression with progression of cancer. Cancer Immunol. Immunother..

[11] Pagès F., Berger A., Camus M., Sanchez-Cabo F., Costes A., Molidor R., Mlecnik B., Kirilovsky A., Nilsson M., Damotte D. (2005). Effector Memory T Cells, Early Metastasis, and Survival in Colorectal Cancer. N. Eng. J. Med..

[12] Sparrow E., Bodman-Smith M. (2020). Granulysin: The attractive side of a natural born killer. Immunol. Lett..

[13] Tong X., Qu X., Wang M. (2021). A Four-Gene-Based Prognostic Model Predicts Overall Survival in Patients with Cutaneous Melanoma. Front. Oncol..

[14] Huang L.P., Lyu S.C., Clayberger C., Krensky A.M. (2007). Granulysin-Mediated Tumor Rejection in Transgenic Mice. J. Immunol..

[15] Al-Wasaby S., de Miguel D., Aporta A., Naval J., Conde B., Martínez-Lostao L., Anel A. (2015). In vivo potential of recombinant granulysin against human tumors. Oncoimmunology.

[16] Al-Wasaby S., Guerrero-Ochoa P., Ibáñez-Pérez R., Soler R., Conde B., Martínez-Lostao L., Anel A. (2021). In vivo potential of recombinant granulysin against human melanoma. Cancer Treat. Res. Commun..

[17] Ibáñez-Pérez R., Guerrero-Ochoa P., Al-Wasaby S., Navarro R., Tapia-Galisteo A., De Miguel D., Gonzalo O., Conde B., Martínez-Lostao L., Hurtado-Guerrero R. (2019). Anti-tumoral potential of a human granulysinbased, CEA-targeted cytolytic immunotoxin. OncoImmunology.

[18] Guerrero-Ochoa P., Aguilar-Machado D., Ibáñez-Pérez R., Macías-León J., Hurtado-Guerrero R., Raso J., Anel A. (2020). Production of a Granulysin-Based, Tn-Targeted Cytolytic Immunotoxin Using Pulsed Electric Field Technology. Int. J. Mol. Sci..

[19] Guerrero-Ochoa P., Ibáñez-Pérez R., Berbegal-Pinilla G., Aguilar D., Marzo I., Corzana F., Minjárez-Sáenz M., Macías-León J., Conde B., Raso J. (2022). Preclinical Studies of Granulysin-Based Anti-MUC1-Tn Immunotoxins as a New Antitumoral Treatment. Biomedicines.

[20] Sanz L., Ibáñez-Pérez R., Guerrero-Ochoa P., Lacadena J., Anel A. (2021). Antibody-Based Immunotoxins for Colorectal Cancer Therapy. Biomedicines.

[21] Shah P., Shende P. (2022). Biomacromolecule-Functionalized Nanoparticle-Based Conjugates for Potentiation of Anticancer Therapy. Curr. Cancer Drug Targets.

[22] De Miguel D., Basáñez G., Sánchez D., Galán P., Marzo I., Larrad L., Naval J., Pardo J., Anel A., Martinez-Lostao L. (2013). Thethering Apo2L/TRAIL to liposomes overcomes chemoresistance of human hematological tumor cells. Mol. Pharm..

[23] De Miguel D., Lemke J., Anel A., Walczak H., Martinez-Lostao L. (2016). Onto better TRAILs for cancer treatment. Cell Death Differ..

[24] Gallego-Lleyda A., De Miguel D., Anel A., Martinez-Lostao L. (2018). Lipid Nanoparticles Decorated with TNF-Related Aptosis-Inducing Ligand (TRAIL) Are More Cytotoxic than Soluble Recombinant TRAIL in Sarcoma. Int. J. Mol. Sci..

[25] De Miguel D., Gallego-Lleyda A., Martinez-Ara M., Plou J., Anel A., Martinez-Lostao L. (2019). Double-Edged Lipid Nanoparticles Combining Liposome-Bound TRAIL and Encapsulated Doxorubicin Showing an Extraordinary Synergistic Pro-Apoptotic Potential. Cancers.

[26] Guo Y., Luan G., Shen G., Wu L., Jia H., Zhong Y., Li R., Li G., Shen Y., Sun J. (2013). Production and characterization of recombinant 9 and 15 kDa granulysin by fed-batch fermentation in Pichia pastoris. Appl. Microbiol. Biotechnol..

[27] Singh R., Letai A., Sarosiek K. (2019). Regulation of apoptosis in health and disease: The balancing act of BCL-2 family proteins. Nat. Rev. Mol. Cell Biol..

[28] Cheong I., Huang X., Thornton K., Diaz L., Zhou S. (2007). Targeting cancer with bugs and liposomes: Ready, aim, fire. Cancer Res..

[29] Ibrahim M., Abuwatfa W., Awad N., Sabouni R., Husseini G. (2022). Encapsulation, Release, and Cytotoxicity of Doxorubicin Loaded in Liposomes, Micelles, and Metal-Organic Frameworks: A Review. Pharmaceutics.

[30] Tarner I., Muller-Ladner U. (2008). Drug delivery systems for the treatment of rheumatoid arthritis. Expert Opin. Drug Deliv..

[31] Hou X., Zaks T., Langer R., Dong Y. (2021). Lipid nanoparticles for mRNA delivery. Nat. Rev. Mater..

[32] Anel A., Gallego-Lleyda A., de Miguel D., Naval J., Martinez-Lostao L. (2019). Role of Exosomes in the Regulation of T-cell Mediated Immune Responses and in Autoimmune Disease. Cells.

[33] Martínez-Lorenzo M.J., Anel A., Gamen S., Monleón I., Lasierra P., Larrad L., Piñeiro A., Alava M.A., Naval J. (1999). Activated human T cells release bioactive Fas ligand and APO2 ligand in microvesicles. J. Immunol..

[34] Naval J., de Miguel D., Gallego-Lleyda A., Anel A., Martinez-Lostao L. (2019). Importance of TRAIL Molecular Anatomy in Receptor Oligomerization and Signaling. Implications for Cancer Therapy. Cancers.

[35] Ejigah V., Owoseni O., Bataille-Backer P., Ogundipe O., Fisusi F., Adesina S. (2022). Approaches to Improve Macromolecule and Nanoparticle Accumulation in the Tumor Microenvironment by the Enhanced Permeability and Retention Effect. Polymers.

[36] Stylianopoulos T. (2013). EPR-effect: Utilizing size-dependent nanoparticle delivery to solid tumors. Ther. Deliv..

[37] Kaufmann S., Vaux D. (2003). Alterations in the apoptotic machinery and their potential role in anticancer drug resistance. Oncogene.

[38] Merino D., Kelly G., Lessene G., Wei A., Roberts A., Strasser A. (2018). BH3-Mimetic Drugs: Blazing the Trail for New Cancer Medicines. Cancer Cell.

[39] Radha G., Raghavan S. (2017). BCL2: A promising cancer therapeutic target. Biochim. Biophys. Acta Rev. Cancer.

[40] Barman H., Walch M., Latinovic-Golic S., Dumrese C., Dolder M., Groscurth P., Ziegler U. (2006). Cholesterol in Negatively Charged Lipid Bilayers Modulates the Effect of the Antimicrobial Protein Granulysin. J. Membr. Biol..

[41] Bosque A., Pardo J., Martínez-Lorenzo M.J., Iturralde M., Marzo I., Piñeiro A., Alava M.A., Naval J., Anel A. (2005). Down-regulation of normal human T cell blast activation: Roles of APO2L/TRAIL, FasL and c- FLIP, Bim or Bcl-x isoform expression. J. Leukoc. Biol..

[42] Martinez-Lostao L., García-Alvarez F., Basáñez G., Alegre-Aguarón E., Desportes P., Larrad L., Naval J., Martínez-Lorenzo M.J., Anel A. (2010). Liposome-Bound APO2L/TRAIL Is an Effective Treatment in a Rabbit Model of Rheumatoid Arthritis. Arthrit. Rheum..

